# Geographical and Epidemiological Characteristics of Sporadic Coronavirus Disease 2019 Outbreaks From June to December 2020 in China: An Overview of Environment-To-Human Transmission Events

**DOI:** 10.3389/fmed.2021.654422

**Published:** 2021-07-16

**Authors:** Maohui Feng, Qiong Ling, Jun Xiong, Anne Manyande, Weiguo Xu, Boqi Xiang

**Affiliations:** ^1^Hubei Key Laboratory of Tumor Biological Behaviors and Hubei Cancer Clinical Study Center, Department of Gastrointestinal Surgery, Wuhan Peritoneal Cancer Clinical Medical Research Center, Zhongnan Hospital of Wuhan University, Wuhan, China; ^2^Department of Anesthesiology, The Second Affiliated Hospital of Guangzhou University of Chinese Medicine, Guangzhou, China; ^3^Hepatobiliary Surgery Center, Union Hospital of Tongji Medical College, Huazhong University of Science and Technology, Wuhan, China; ^4^School of Human and Social Sciences, University of West London, London, United Kingdom; ^5^Department of Orthopedics, Tongji Hospital of Tongji Medical College, Huazhong University of Science and Technology, Wuhan, China; ^6^School of Public Health, Rutgers University, New Brunswick, NJ, United States

**Keywords:** COVID-19 disease, cold-chain food, scientific protective measures, severe acute respiratory syndrome coronavirus 2, environment-to-human transmission

## Abstract

China quickly brought the severe acute respiratory syndrome coronavirus 2 under control during the early stage of 2020; thus, this generated sufficient confidence among the public, which enabled them to respond to several sporadic coronavirus disease 2019 outbreaks. This article presents geographical and epidemiological characteristics of several sporadic coronavirus disease 2019 outbreaks from June to December 2020 in China. The data show that the coronavirus disease may be transmitted by imported cold-chain food and international exchange, and this viewpoint deserves our great attention.

## Introduction

Severe acute respiratory syndrome coronavirus 2 (SARS-CoV-2) was reported in China toward the end of December 2019. It has spread across the globe, causing a pandemic. The World Health Organization called it the SARS-CoV-2 pandemic on March 11, 2020. By the end of December 31, 2020, coronavirus disease 2019 (COVID-19) had spread to 218 countries with 83,260,611 cases and 1,816,120 deaths. A growing body of studies indicates that rapid dissemination of SARS-CoV-2 occurs mainly through oral saliva (Flugge's droplets) and airborne routes, as well as *via* nasal and lachrymal passages or fecal contacts (1–4). It is well-known that Chinese anti-contagion strategies for the COVID-19 pandemic were especially effective during the early stage of 2020 (5, 6). This affords a unique opportunity to monitor the local recurrence of COVID-19 cases and adopt effective preventive measures and remedies for the complete control of COVID-19.

For the initial sporadic COVID-19 outbreaks, the urgent concerns for public health required a deep understanding of its epidemiology, transmissibility, and control. Recent reports have focused attention on the importance of social practices, including social distancing and wearing of masks in anti-contagion strategies for the COVID-19 (7, 8). Based on potential transmission conditions of SARS-CoV-2, no effective methods have been found to prevent the spread of the COVID-19 epidemic (9, 10), apart from emphasizing social distancing, antibody testing for infected cases, identifying asymptomatic carriers, isolating infected patients, and using COVID-19 vaccines. Saving lives and livelihoods with a combination of public health measures and vaccination—not one or the other—are the keyway out to the COVID-19 pandemic. Given the potential role of the environment-to-person transmission spread of COVID-19 (2), the objectives of the study aim to present geographical and epidemiological characteristics of several sporadic COVID-19 outbreaks from June to December 2020 in China. Our data show that the coronavirus disease may be transmitted by imported cold-chain food and international exchange, and this viewpoint deserves our great attention. China's experience in containing the spread of the new coronavirus could serve as a lesson for other countries now facing the COVID-19 pandemic.

## Methods

At the beginning of June 1, 2020, we prospectively focused on the COVID-19 epidemic data from the Chinese Center for Disease Control and Prevention every day. Once we received the new report of a confirmed or asymptomatic case in China, we tracked this epidemic, collected its epidemiological characteristics from announcements by the local Health Commission, and presented a descriptive analysis of the occupational characteristics and management measures of sporadic outbreaks of COVID-19 (from June to December 2020).

We also compiled publicly available data from the China Center for Disease Control and Prevention, National Health Commission in Beijing, Qingdao, Dalian, and World Health Organization. In addition, we searched reports from the media and announcements from the local Municipal Health Commissions related to the cold-chain food transmission infection from June to December 2020. All confirmed cases had to undergo a COVID-19 nucleic acid polymerase chain reaction test. Analyses included the distribution of the sporadic outbreak in China, imported frozen product chains in Beijing, live virus in Qingdao, and other results. We also collected literature using the PubMed database and Cochrane Library from June 1, 2020, to December 31, 2020. Search terms included “cold chain food” and “novel coronavirus” or “COVID-19” or “2019-nCoV” using the Boolean operators “AND” and “OR.”

## Results

### Regional Distribution of the Sporadic Outbreaks With Coronavirus Disease 2019 From June to December 2020

**Figure 1:**
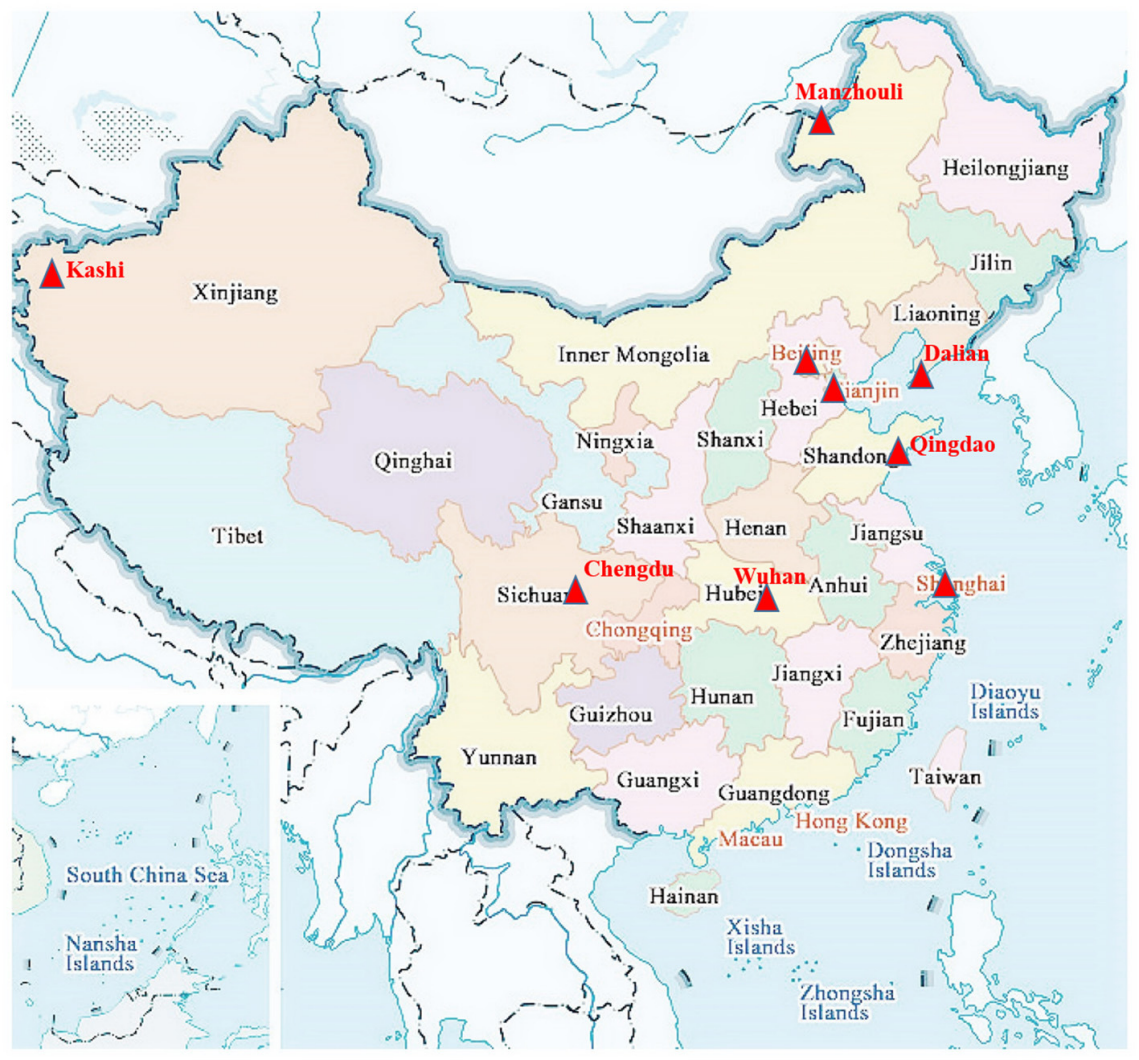
Geographical epidemiological characteristics of eight cities during the sporadic COVID-19 outbreaks in China. Dalian, Tianjin, Qingdao, and Shanghai are coastal cities, and Kashi and Manzhouli are border cities.

The regional distribution of the sporadic COVID-19 outbreaks from June to December 2020 is shown in Figure 1. Most of these cities are located in coastal or border areas; for example, Dalian, Tianjin, Qingdao, and Shanghai are coastal cities, whereas Kashi and Manzhouli are border cities.

### Epidemiological Characteristics of the Sporadic Coronavirus Disease 2019 Outbreaks From June to December 2020

**Figure 2:**
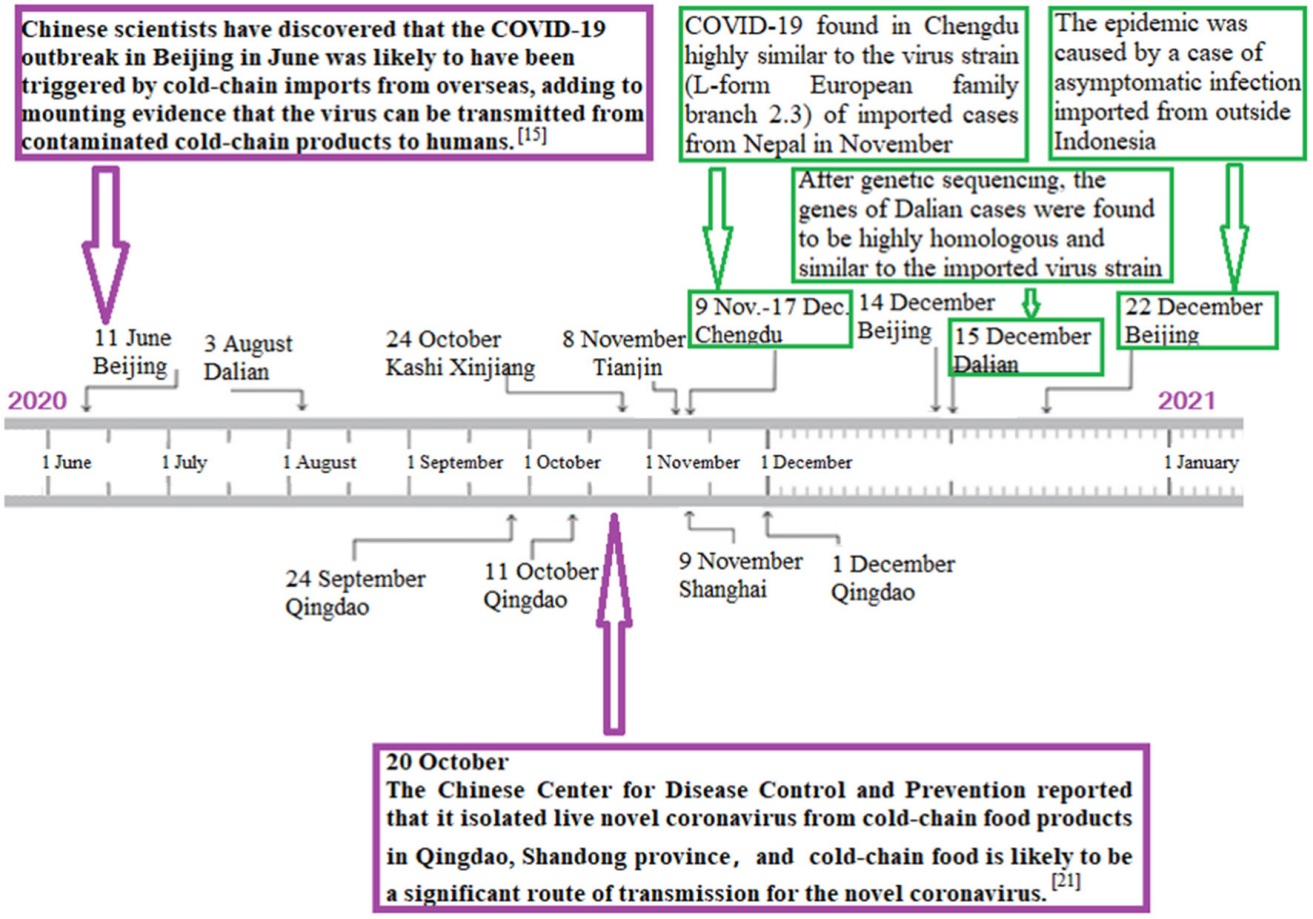
Graph's x-axis (dates from June 1 to December 31, 2020) is used as a timeline of key events and dynamic profile of cold-chain environment-to-human transmission during sporadic COVID-19 outbreaks in China. In Xinfadi of Beijing, Dalian, Qingdao, Kashi, Tianjin, and Shanghai, polymerase chain reaction on environmental swab samples (i.e., frozen food package) related to imported cold-chain food were positive for SARS-CoV-2. In Qingdao, live SARS-CoV-2 was isolated from imported frozen cod outer package's surface, showing that there exists cold-chain environment-to-human transmission.

Cities with sporadic COVID-19 outbreaks are reported in Figure 2 and Table 1. In many cities or areas, such as Xinfadi of Beijing, Dalian, Qingdao, Kashi, Tianjin, and Shanghai, the polymerase chain reaction on environmental swab samples (i.e., frozen food package) related to imported cold-chain food tested positive for SARS-CoV-2. In Qingdao, live SARS-CoV-2 were isolated from the imported frozen cod outer package's surface (Figure 2), showing a possibility of cold-chain environment-to-human transmission.

**Table 1:** Epidemiological data of sporadic COVID-19 outbreaks from June to December 2020 in China.

**City**	**Date of first case in local fresh outbreak**	**Confirmed cases**	**Asymptomatic infection cases**	**Source of infection**	**Database**
Beijing	June 11	335		Cold seafood	Link 1; Link1.2
	December 14	3		Close contact with confirmed patients from overseas	Link 2
Dalian	July 9	118	26	Imported frozen food	Link 3
	December 15	41	28	Cold-chain transportation	Link 4
Qingdao	October 11	2	12	Imported frozen food	Link 5
	December 1	0	2	Cold-chain food	Link 6
Kashi	October 24	78	350	Imported aviation container	Link 7
Tianjin	November 8	10		Imported frozen food	Link 8
Shanghai	November 9	3		Overseas aviation container	Link 9
Manzhouli	November 21	23	2	Close contact with confirmed patients from overseas	Link 10
Chengdu	December 7	14		Garbage from imported cases	Link 11

*1 http://www.xinhuanet.com/politics/2020-06/14/c_1126112688.htm. 1.2 https://sciencespeaksblog.org/2020/06/14/covid-19-in-beijing-a-new-outbreak-linked-with-large-market-xinfadi/*.

*2 https://www.bjcdc.org/article/65306/2020/12/1607995074196.html*.

*3 http://dlyj.dl.gov.cn/c/2020-07-26/493164.shtml*.

*4 http://dl.bendibao.com/news/20201218/64440.shtm*.

*5 https://news.sina.com.cn/o/2020-10-12/doc-iivhvpwz1479996.shtml*.

*6 http://dsyj.qingdao.gov.cn/n28356085/n32568340/n32568356/210111111034333787.html*.

*7 https://finance.sina.com.cn/roll/2020-10-26/doc-iiznctkc7620995.shtml*.

*8 https://news.sina.com.cn/c/2020-11-09/doc-iiznctke0341075.shtml*.

*9 http://society.people.com.cn/n1/2020/1109/c1008-31924581.html*.

*10 https://finance.sina.com.cn/tech/2020-11-22/doc-iiznctke2651002.shtml*.

*11 https://sichuan.scol.com.cn/ggxw/202012/58005103.html*.

### Geographical Epidemiological Characteristics of Coronavirus Disease 2019 Outbreak in Beijing July 2020

**Figure 3:**
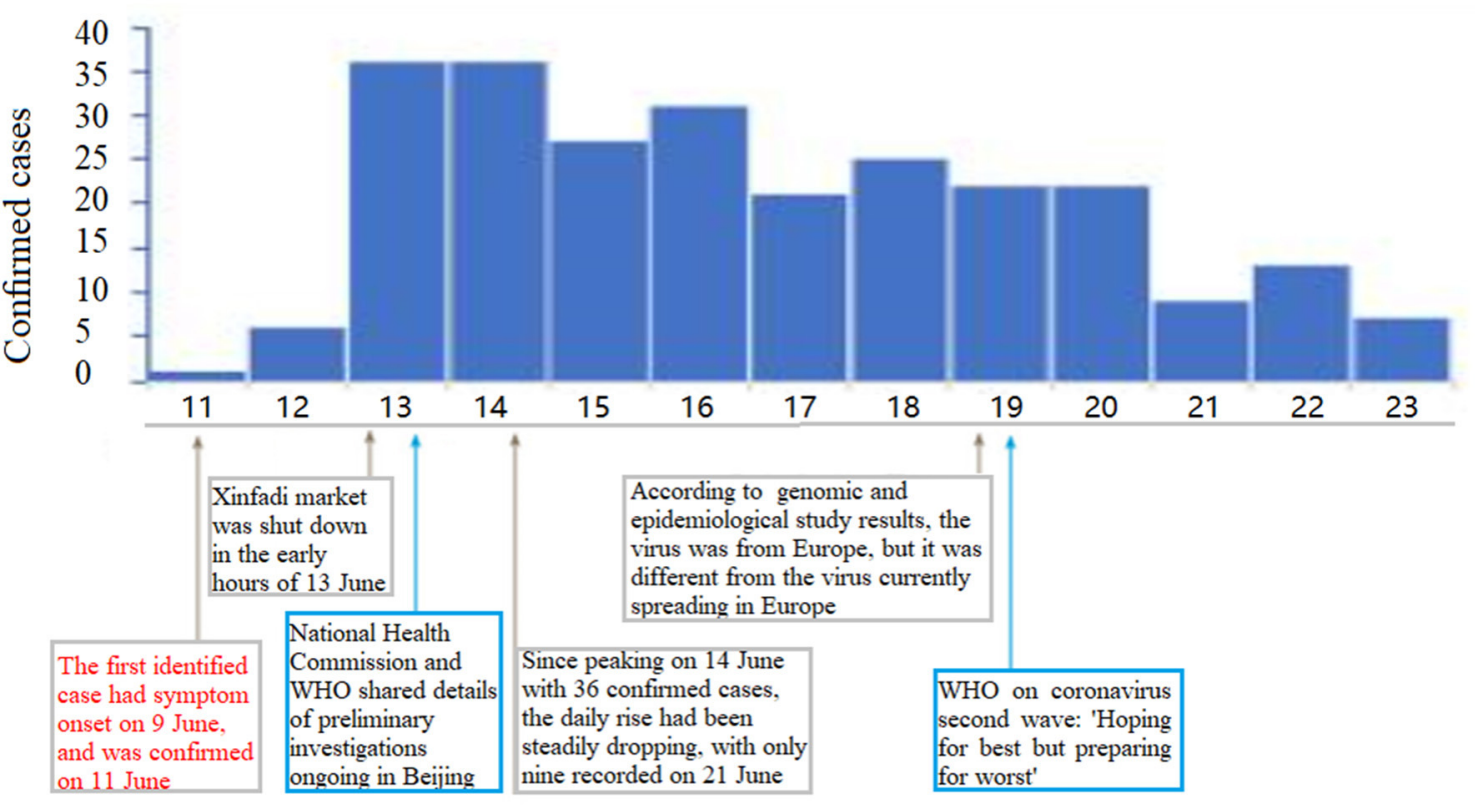
Graph's x-axis (dates from June 11 to 23, 2020) is used as a timeline of key events and dynamic profile during COVID-19 outbreak. Profiles of confirmed cases are shown in graph's y-axis. First case in Beijing's fresh outbreak was recorded on June 11. Inset shows that daily rise had been steadily dropping, with only nine recorded on Jun 21, since peaking on June 14 with 36 confirmed cases.

As of June 23, 2020, the Chinese National Health Commission reported 256 confirmed COVID-19 cases in Beijing. The geographical distribution of these hospitals is shown in Supplementary Table 1 and Figure 3. The first confirmed COVID-19 case in Beijing's fresh outbreak was recorded on June 11 (Supplementary Table 1). Figure 3 describes the chronology of the confirmed COVID-19 cases. The inset illustrates that the daily rise had been steadily dropping, with only nine recorded on June 21, since peaking on June 14 with 36 confirmed cases (Figure 3).

**Figure 4:**
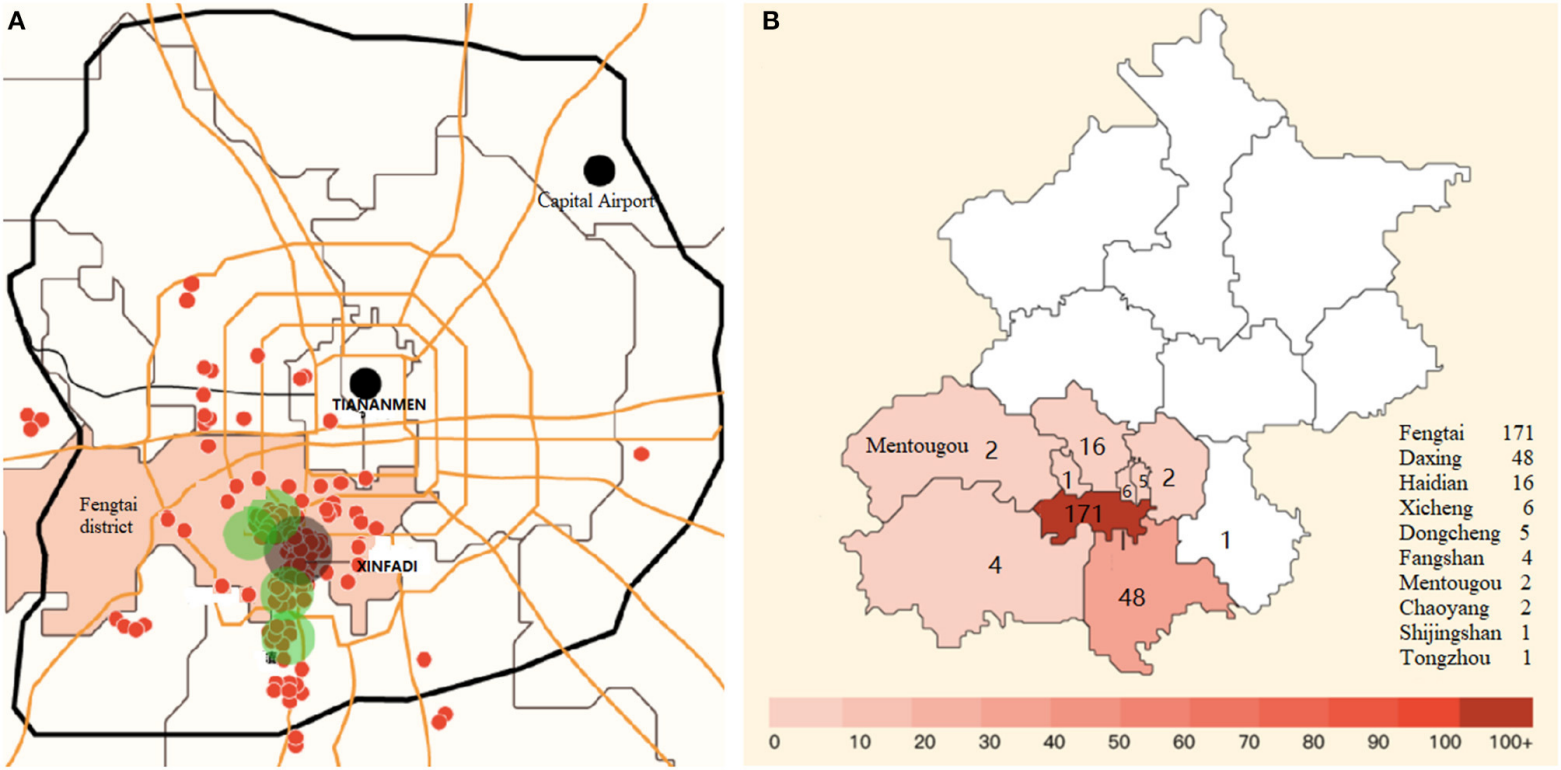
Geographical epidemiological characteristics of confirmed cases in Beijing city. **(A)** Geographical distribution of all confirmed cases. **(B)** Spatial distribution of confirmed cases. Proportion of confirmed cases in Fengtai district was 66.8% of overall cases in Beijing. Red dots represent spatial distribution of confirmed cases, and green dots stand for the top five concentrated communities of confirmed cases.

The overall spatial distribution of confirmed cases in Beijing is presented in Figure 4. The proportion of confirmed cases in Fengtai district was 66.8% of the overall cases in Beijing. The overall male-to-female ratio in Beijing was 1.3:1.

### Beijing and Wuhan's Responses to Coronavirus Disease 2019 Outbreak

The comparison between Wuhan and Beijing's outbreaks and response is demonstrated in Supplementary Table 2. In Beijing city, a new outbreak had been reported with an epidemiological link to the largest food/seafood market, called “Xinfadi.” Cold-chain food contamination was regarded as the possible origin of COVID-19 resurgence in Beijing (Figure 2), as was the source of the Wuhan outbreak linked initially to the Huanan Seafood market, which closed on January 1, 2020.

As in the Wuhan market, some environmental samples from the Xinfadi market had tested positive. The Fengtai district, where the Xinfadi market is located, an area of the Chinese capital Beijing, had been put on a “wartime emergency mode” and under strict lockdown measures to stop the COVID-19 epidemic after the city's first coronavirus cases were detected in more than 50 days. Lockdowns had been imposed in 11 nearby neighborhoods and several other food or seafood markets in Beijing. All sports events and tour groups had also been shut down, and school openings had been delayed, whereas 10,000 market staff were tested for COVID-19.

## Discussion

It is well established that anti-contagion strategies for virus transference have become a matter of essential routine in everyday life. Our results show that sporadic COVID-19 outbreaks from June to December 2020 in China were linked to the cold-chain environment-to-human transmission. Many scientists have investigated coronavirus persistence on a plastic carrier under a cold-chain environment or different temperature conditions (2, 11–13) and assessed the evidence and risk factors on food contamination and foodborne transmission of SARS-CoV-2 (14).

Pang et al. (15) reported that environmental swab samples related to imported cold-chain food tested nucleic acid-positive for SARS-CoV-2 in Xinfadi, suggesting that cold-chain food contamination could be the possible origin of COVID-19 resurgence in Beijing. As confirmed cases of a novel coronavirus SARS-CoV-2 surged around Fengtai district in Beijing, with worrisome speed on June 2020, the focus was on the Xinfadi wet market, which was the site of a superspreader event—the Beijing's COVID-19 resurgence. The Huanan seafood market in Wuhan had also been widely considered to be the source of the SARS-CoV-2-induced COVID-19 pandemic (5). A description of the Wuhan clinical cases published in *The Lancet* showed that, in total, 28 of the 41 cases were linked to the marketplace (16), suggesting that most cases had links to the seafood market, which was closed on January 1, 2020.

The sporadic COVID-19 outbreaks in Beijing (June 2020) were highly similar to those in Wuhan. The comparison of the COVID-19 pandemic between Wuhan and Beijing can be found in Supplementary Table 2. As with the Wuhan Huanan wet market in December 2019, the spread of the SARS-CoV-2 in the Beijing Xinfadi wet market in June 2020 could have occurred in two ways: animal to person and person to person. Because a significant number of the initial cases did not have contact with Huanan wet market in the Wuhan SARS-CoV-2 pandemic (17), this casts doubt on the singular event of animal-to-human transmission in the initial outbreak. Data from Supplementary Table 1 demonstrate that most cases were linked to the Xinfadi market, and there is evidence of human-to-human transmission. As of June 13, 2020, 40 environmental samples from Xinfadi Market had tested SARS-CoV-2-positive (18). An article from the *American National Public Radio* detailed the industry's first major outbreak onboard a huge vessel with an onboard fish processing factory, and Seattle-based American seafood confirmed that 92 crew from its American Dynasty ship had tested positive for COVID-19, nearly three-fourths of the 126 people onboard (19). Moreover, one SARS-CoV-2-positive patient in France in late December 2019 was identified as close contact to his wife with a seafood salesperson (20). Based on the epidemiological survey data mentioned earlier, the unleashing of the second wave of SARS-CoV-2 in Beijing might come from imported frozen chain food products.

Beijing's COVID-19 resurgence was similar to the one in Wuhan during the early period of the outbreak, but the authorities' responses were different (Supplementary Table 2). Wuhan City went into shut down to stop the spread of the coronavirus from January 23 to April 8, and it had exceptionally long coronavirus lockdowns compared with other neighboring regions and cities. To stop the virus from spreading from Wuhan to the nation, authorities banned all travel out of Wuhan, prompting all regular Wuhan flights to be canceled.

In the COVID-19 epidemic of Beijing's Xinfadi market, the live virus had not been isolated due to the low nucleic acid concentration of the salmon samples tested. Liu et al. (21) showed successful isolation of the live SARS-CoV-2 from the imported frozen cod package surface in Qingdao, suggesting that cold-chain transportation in the frozen food industry might have triggered a recurrence of COVID-19 cases. It is worth mentioning that because no viral sequence clustering and homology has been provided in this report, any statement about the transmission origin of the virus remains speculative.

## Conclusion

Here, we retrospectively reported specific patterns of sporadic COVID-19 outbreaks from June to December 2020 in China. Chinese authorities fully invested in having an aggressive track, test, and trace surveillance system to minimize the resurgence of the possible environment-to-human transmission and reduce the likelihood of the virus rebound. China's experience in containing the spread of the new coronavirus could serve as a lesson for other countries now facing the COVID-19 pandemic.

## Data Availability Statement

The original contributions presented in the study are included in the article/Supplementary Material, further inquiries can be directed to the corresponding author/s.

## Author Contributions

MF, QL, and BX: data collection. JX, WX, AM, and BX: data interpretation and writing. All authors contributed to the article and approved the submitted version.

## Conflict of Interest

The authors declare that the research was conducted in the absence of any commercial or financial relationships that could be construed as a potential conflict of interest.
