# Supplementary material for: Geographical and Epidemiological Characteristics of Sporadic Coronavirus Disease 2019 Outbreaks From June to December 2020 in China: An Overview of Environment-To-Human Transmission Events
Source: Front Med (Lausanne). 2021 Jul 16;8:654422. doi: 10.3389/fmed.2021.654422 (PMC8322611; doi:10.3389/fmed.2021.654422)
Supplement: Supplementary file 1 [file Table_1.docx]

**Table 1 The epidemiological data for confirmed cases on the early periods of second wave coronavirus outbreaks in Beijing**

| patient No. | Age (years) |  | Sex | Epidemiology History (Have been to XFD market) | SARS-CoV2 RT-PCR result | symptom date | Days from symptom onset to nasopharyngeal swab | Symptoms at disease onset | Epidemiology data | Confirmed date |
| --- | --- | --- | --- | --- | --- | --- | --- | --- | --- | --- |
| 1 | 52 |  | M | 6/3 | Positive | 6/6 | 5 | Fever | market worker | 6/11 |
| 2 | 25 |  | M | 6/5 | Positive | 6/9 | 3 | Fever, cough | Covid-19 investigator | 6/12 |
| 3 | 37 |  | M | 6/7 | Positive | 6/9 | 3 | Throat itch, cough | bought groceries | 6/12 |
| 4 | 38 |  | M | 6/5 | Positive | 6/9 | 3 | fever, headache | bought frozen shrimp and crawfish | 6/12 |
| 5 | 50 |  | M | 6/4, 6/5 | Positive | 6/6 | 6 | fever, cough | bought lamb | 6/12 |
| 6 | 35 |  | M | 6/4 | Positive | 6/10 | 2 | cough | bought lamb | 6/12 |
| 7 | 27 |  | F | residence staff | Positive | 6/9 | 3 | cough | market worker | 6/12 |
| 8 | 35 |  | M | residence staff | Positive | 6/8 | 4 | fever, cough，running nose, fatigue, headache, body soreness | market worker | 6/12 |
| 9 | 34 |  | F | close contact with XFD | Positive | 6/8 | 4 | fatigue, muscular soreness | close contact with XFD | 6/12 |
| 10 | 58 |  | F | Went to XFD almost every day | Positive | 6/7 | 4 | fever, chills, running nose | bought groceries | 6/11 |
| 11 | 56 |  | M | 6/4 | Positive | 6/9 | 1 | Muscular soreness, fever | bought aquatic products, fruits | 6/10 |
| 12 | 51 |  | M | 6/4 | Positive | 6/7 | 5 | Dizziness, fatigue, pharyngalgia | bought aquatic products, fruits | 6/12 |
| 13 | 46 |  | F | residence staff | Positive | 6/10 | 2 | dizziness, fever, nausea | market worker | 6/12 |
| 14 | 32 |  | M | residence staff | Positive | - | - | None Symptom | market worker | 6/12 |
| 15 | 28 |  | F | residence staff | Positive | - | - | None Symptom | market worker | 6/12 |
| 16 | 43 |  | F | residence staff | Positive | 6/9 | 3 | body soreness, chills, shiver, diarrhea | market worker | 6/12 |
| 17 | 44 |  | M | residence staff | Positive | 6/10 | 2 | throat hurt, cough | market worker | 6/12 |
| 18 | 41 |  | M | 6 times to XFD from 5/28-6/11 | Positive | 6/11 | 1 | fever, headache | bought beef and lamb | 6/11 |
| 19 | 43 |  | M | residence staff | Positive | 6/4 | 8 | headache, fatigue | market worker | 6/12 |
| 20 | 38 |  | F | residence staff | Positive | - | - | None Symptom | market worker | 6/12 |
| 21 | 42 |  | F | residence staff | Positive | - | - | None Symptom | market worker | 6/12 |
| 22 | 32 |  | M | residence staff | Positive | 6/11 | 1 | Throat ache | market worker | 6/12 |
| 23 | 54 |  | M | residence staff | Positive | 6/8 | 4 | chills, fever | market worker | 6/12 |
| 24 | 56 |  | M | 6/3 | Positive | 6/5 | 7 | chills, headache, fever | bought groceries | 6/12 |
| 25 | 27 |  | M | residence staff | Positive | 6/11 | 1 | cough, chills, expectoration | market worker | 6/12 |
| 26 | 38 |  | M | residence staff | Positive | 6/10 | 2 | cough, throat itch, sneeze | market worker | 6/12 |
| 27 | 28 |  | M | residence staff | Positive | 6/10 | 2 | fever | market worker | 6/12 |
| 28 | 27 |  | F | residence staff | Positive | 6/4 | 8 | running nose, sneeze, fatigue, muscular soreness | market worker | 6/12 |
| 29 | 35 |  | F | residence staff | Positive | 6/11 | 1 | dizziness, chills | market worker | 6/12 |
| 30 | 49 |  | F | residence staff | Positive | - | - | None Symptom | market worker | 6/12 |
| 31 | 46 |  | F | 6/6 | Positive | 6/11 | 1 | muscular soreness, fever | bought groceries | 6/12 |
| 32 | 31 |  | F | Her husband had multiple exposures at XFD, she doesn’t | Positive | 6/8 | 4 | fever, fatigue, sneeze, running nose, diarrhea. | - | 6/12 |
| 33 | 61 |  | F | residence staff | Positive | 6/10 | 2 | dry throat, fever | market worker | 6/12 |
| 34 | 46 |  | F | residence staff | Positive | - | - | Insomnia | market worker | 6/12 |
| 35 | 50 |  | F | residence staff | Positive | 6/10 | 2 | dry throat | market worker | 6/12 |
| 36 | 33 |  | F | residence staff | Positive | - | - | None Symptom | market worker | 6/12 |
| 37 | 29 |  | M | residence staff | Positive | - | - | None Symptom | market worker | 6/12 |
| 38 | 25 |  | M | residence staff | Positive | - | - | None Symptom | market worker | 6/12 |
| 39 | 34 |  | F | residence staff | Positive | - | - | None Symptom | market worker | 6/12 |
| 40 | 50 |  | F | residence staff | Positive | - | - | None Symptom | market worker | 6/12 |
| 41 | 38 |  | F | residence staff | Positive | - | - | None Symptom | market worker | 6/12 |
| 42 | 35 |  | F | residence staff | Positive | - | - | None Symptom | market worker | 6/12 |
| 43 | 48 |  | M | residence staff | Positive | 6/13 | -1 | headache | market worker | 6/12 |
| 44 | 27 |  | M | residence staff | Positive | - | - | None Symptom | market worker | 6/12 |
| 45 | 42 |  | M | residence staff | Positive | - | - | None Symptom | market worker | 6/12 |
| 46 | 47 |  | F | 6/3 | Positive | 6/11 | 1 | Fever | bought groceries | 6/12 |
| 47 | 57 |  | M | - | Positive | 6/10 | 3 | Intolerance of cold | - | 6/13 |
| 48 | 37 |  | M | 6/4 | Positive | 6/8 | - | Feeling uncomfortable | bought groceries | - |
| 49 | 35 |  | F | - | Positive | 6/12 | 0 | fever | close contact with patient No. 48 | 6/12 |
