# Supplementary material for: Geographical and Epidemiological Characteristics of Sporadic Coronavirus Disease 2019 Outbreaks From June to December 2020 in China: An Overview of Environment-To-Human Transmission Events
Source: Front Med (Lausanne). 2021 Jul 16;8:654422. doi: 10.3389/fmed.2021.654422 (PMC8322611; doi:10.3389/fmed.2021.654422)
Supplement: Supplementary file 2 [file Table_2.docx]

**Table 2 The analysis of COVID-19 pandemic between Wuhan and Beijing**

|  | COVID-19 pandemic in Wuhan | COVID-19 pandemic in Beijing |
| --- | --- | --- |
| The first identified case | symptom onset on December 8, 2019, and confirmed on December 10, 2019 | symptom onset on June 9, 2020, and confirmed on June 11, 2020 |
| The possible source of epidemiological link | Huanan Seafood market | Xinfadi food/seafood market, in the Beijing city’s south-western Fengtai district |
| Information of the World Health Organization | Wuhan authorities informed the World Health Organization on December 31, 2019 about the unknown, pneumonialike illness that would later be identified as COVID-19. | National Health Commission and Beijing Health Commission briefed WHO’s China country office, to share details of preliminary investigations ongoing in Beijing on June 13, 2020. |
| Environmental samples from two markets | SARS-CoV-2-positive | SARS-CoV-2-positive |
| Closing date of wet market | January 1, 2020 | June 13, 2020 |
| Traced exposures | there is a strong indication that untraced exposures other than the one in the epidemiologically linked seafood market in Wuhan have occurred. | Only Xinfadi food Seafood/market |
| Date of city lockdown | Wuhan city shut down on January 23, 2020, and another 15 cities down on January 24, 2020. | Xinfadi market and surrounding neighbourhoods had been adopted lockdown measures on June 13, 2020. |
| strict lockdown measures | China’s coronavirus outbreak was brought under control through some of the world’s strictest lockdown measures after the disease was detected in the city of Wuhan. | The Fengtai district was in a “wartime emergency mode”, and hundreds of police had entered the locked-down facility. Nearby transport links and schools had been closed. Right across Beijing sport had been cancelled and major public facilities were pulling down the shutters. |
| COVID-19 testing | Limited detection during early periods of COVID-19 pandemic | When Beijing's second outbreak took off, the city immediately ramped up its COVID-19 testing. |
| A messaging APP as contact tracer | Wuhan turned a messaging APP into a contact tracer during late stage of COVID-19 pandemic | Local authorities across China have built programs into the messaging app. It tracked your movements and assigns you a color code based on where you've been.  In parts of Beijing people could not enter buildings unless they had a green rating on China's contact tracing APP. A green rating means you could move around without restrictions. Anyone with an orange or red rating was not allowed to travel. |
| The possible source of SARS-CoV-2 | unknown | It could have been hidden in imported frozen food products, or it was lurking in some dark and humid environment such as Xinfadi market, with the environment not having been disinfected or sterilized. |
| Results | The tables have turned after February 11, 2020 | Since peaking on June 14 with 36 confirmed cases, the daily rise had been steadily dropping, with only nine recorded on June 21. |
